# Integrated Transcriptome and Metabolome Analysis of the Porcine Small Intestine During Weaning

**DOI:** 10.3390/genes16070727

**Published:** 2025-06-22

**Authors:** Jung Woong Yoon, Sangsu Shin, Tae Hyun Kim, Sang In Lee

**Affiliations:** 1 Department of Animal Science and Biotechnology, Kyungpook National University, Sangju-si 37224, Gyeongsangbuk-do, Republic of Korea; kizx789@knu.ac.kr (J.W.Y.); sss@knu.ac.kr (S.S.); 2 Research Institute for Innovative Animal Science, Kyungpook National University, Sangju-si 37224, Gyeongsangbuk-do, Republic of Korea; 3 Department of Animal Science, The Pennsylvania State University, University Park, PA 16802, USA

**Keywords:** porcine small intestine, weaning, gene expression profiling, metabolome analysis

## Abstract

Background/Objectives: Intestinal dysfunction during weaning in piglets causes declines in growth through hindered absorption capacity and intestinal barrier function, equating to economic losses for the porcine industry. Established strategies for mitigating these negative issues are currently lacking. Methods: We evaluated biomolecular alterations induced by weaning stress through gene expression profiling and metabolome analysis using intestinal samples collected from piglets before weaning, 1 week after weaning, and 2 weeks after weaning. Results: We identified 701 differentially expressed genes related to weaning stress, representing the enrichment of Gene Ontology and Kyoto Encyclopedia of Genes and Genomes (KEGG) pathways associated with immune response; inflammatory response; cell proliferation; cell adhesion; and carbohydrate, lipid, and calcium ion binding. In the metabolome analysis, ABC transporter; purine, pyrimidine, and Gly-Ser-Thr metabolisms; and the urea cycle were clustered as enriched KEGG pathways. Our results suggest that energy metabolism, including protein metabolism, is involved in the repair of the structural damage occurring in the intestine during weaning. Conclusions: This study highlights the importance of integrated analyses synthesizing molecular and metabolic mechanisms in elucidating complex biological responses and provides insights into markers that can be used to develop strategies for mitigating weaning stress in the porcine industry.

## 1. Introduction

With global population growth and the rapid development of countries’ infrastructures, the consumption of meats, including beef, pork, and chicken, is accelerating. Among livestock, pigs have a high production efficiency, high reproductive capacity, produce high-quality protein, and have a wide utilization, which makes pig husbandry increasingly important in the livestock industry [1,2,3]. However, pigs face various challenges during rearing, such as parturition [4], heat [5], transport [6], and weaning stress [7]. As a stressor, weaning, in particular, is related to physiological, nutritional, environmental, and emotional transitions [8]. During the weaning period, pigs are vulnerable to disorders that adversely affect intestinal homeostasis, the immune system, feed efficiency, and weight gain, resulting in economic losses to the porcine industry [9,10]. Thus, management strategies that reduce weaning’s adverse effects on pig health and, hence, the porcine industry are necessary.

The small intestine plays a vital bifunctional role in health, performing nutrient absorption and providing a defensive boundary against harmful biological factors [11,12]. The weaning transition is an unavoidable challenge to the maintenance of proper intestinal function, as it causes physiological alterations to the digestive and immune systems, including changes to activated metabolic enzymes, immunocytes, inflammatory cytokines, and immunoglobulins, and facilitates the invasion of pathogens, triggered by exposure to novel feed types and other exogenous/endogenous factors [13,14]. Weaning-mediated alterations in the small intestine induce structural and morphological damage, such as shortened villi and elongated crypts. If this damage does not heal properly, it can cause dehydration, diarrhea, immune dysregulation, intestinal barrier disruption, reduced weight gain, and, in severe cases, death [15,16]. These post-weaning adverse intestinal effects continue to be obstacles in the porcine industry, and research improving our understanding of the physiological interactions of the intestine is needed to maximize pig productivity.

As genome research expands, so does our knowledge of how, when, and to what extent genes are expressed and of how gene expression affects the function and condition of organisms [17,18,19]. This research has shown that diverse molecular mechanisms organize enormous biological networks. Among these networks, metabolism, including processes like glycolysis, lipid synthesis, and peptide transport, is directly influenced by gene expression and plays a vital role in maintaining homeostasis [20,21]. Furthermore, exogenous and endogenous pathogens, including bacteria, viruses, and other microorganisms, utilize host metabolites to enhance their own survival and virulence, thereby exacerbating diseases and furthering infections [22,23,24]. Accurately predicting and preventing weaning transition-mediated adverse effects is difficult due to the complicated interactions of various genes and metabolites [25,26]. Nevertheless, greater biological network understanding and research is important in order to minimize the adverse effects of the weaning transition on the porcine industry.

We aimed to explore alterations in gene and metabolite expression in the small intestine during weaning. Our results will benefit the porcine industry by helping provide a theoretical basis for understanding weaning’s negative effects on pig intestinal health and developing mitigation strategies.

## 2. Materials and Methods

### 2.1. Animals and Sampling for Gene Expression Profiling and Metabolite Analysis

A total of 9 crossbred pigs [(Yorkshire × Landrace) × Duroc] were used for extracting intestinal tissues according to the weaning period, with collections representing three periods: before weaning, 1 week after weaning, and 2 weeks after weaning. At 28 d, all pigs were weaned and housed in an environmentally controlled room with a slatted plastic floor and self-feeder and nipple waterer to allow ad libitum access to feed and water throughout the experimental period. Feed was a corn–soybean meal-based diet. During the feeding trial, three piglets per treatment were sedated with xylazine and ketamine and euthanized with an overdose of pentobarbital administered via an ear vein. The abdominal cavity of the piglets was opened, and intestinal samples were removed from the small intestine.

For gene expression analysis, total RNA was isolated from intestinal tissue samples using TRIzol reagent (Invitrogen, Carlsbad, CA, USA). For metabolite analysis, samples were extracted using two protocols. First, the samples were immersed in 750 µL of 50% acetonitrile in water (*v*/*v*) containing internal standards (20 µM) and homogenized using a homogenizer, with five cycles at 1500 rpm for 120 s. Subsequently, an equal volume of 50% acetonitrile in water (*v*/*v*) was added, and 400 µL of supernatant was collected after filtration through a 5 kDa cut-off filter (Ultrafree-MC-PLHCC, Human Metabolome Technologies, Yamagata, Japan), removing macromolecules. The filtrate was then concentrated through centrifugation and resuspended in 50 µL of ultrapure water immediately before measurement. In the second protocol, samples were immersed in 500 µL of 1% formic acid in acetonitrile (*v*/*v*) containing internal standards (20 µM) and homogenized as described above. After adding 167 µL of Milli-Q water, the mixture was homogenized again and centrifuged at 2300× *g* and 4 °C for 5 min. The supernatant was collected, and the remaining pellet was re-extracted with an additional 500 µL of 1% formic acid in acetonitrile and 167 µL of Milli-Q water. After repeating the homogenization and centrifugation, the collected supernatants were combined and filtrated through a 3 kDa cut-off filter (Nanocep 3K Omega, Pall Corporation, Ann Arbor, MI, USA) to remove proteins and then further purified using a phospholipid removal column (Hybrid SPE Phospholipid 55261-U, Supelco, Bellefonte, PA, USA). The final filtrate was desiccated and resuspended in 100 µL of 50% isopropanol in Milli-Q water (*v*/*v*) immediately before measurement.

### 2.2. Gene Expression Profiling

Total RNA samples were assessed for quality using an Agilent 2100 Bioanalyzer System (Agilent Technologies, Amstelveen, The Netherlands), and the library was constructed using the Clontech SMARTer Stranded RNA-Seq Kit (Takara Bio USA, Inc., Mountain View, CA, USA) according to the manufacturer’s protocol. Sequencing was conducted on the Illumina platform using paired-end 100 bp (PE100) reads. Differentially expressed genes (DEGs) were analyzed using an Excel-based differential expression analysis, and functional annotations for the DEGs were identified through Gene Ontology (GO) and Kyoto Encyclopedia of Genes and Genomes (KEGG) pathway analyses using the Database for Annotation, Visualization, and Integrated Discovery.

### 2.3. Metabolite Analysis

Metabolites were identified and measured using capillary electrophoresis time-of-flight mass spectrometry (CE-TOFMS) in the cation and anion modes and liquid chromatography time-of-flight mass spectrometry (LC-TOFMS) in the positive and negative modes. The peaks detected in the CE-TOFMS and LC-TOFMS analyses were extracted using automatic integration software (MasterHands ver. 2.17.1.11, developed at Keio University) in order to obtain peak information, including *m*/*z* ratios, peak areas, and migration times (MTs) for CE-TOFMS or retention times (RTs) for LC-TOFMS. The peak area was then converted to relative peak area using the equation. The peak detection limit was determined based on the signal–noise ratio: S/N = 3. Putative metabolite identifications were then assigned to peaks from HMT’s standard library and the Known-Unknown peak library on the basis of their *m*/*z* value and MT or RT. The tolerances were ±0.5 min for MTs and ±0.3 min for RTs and ±10 ppm for CE-TOFMS *m*/*z* values and ±20 ppm for LC-TOFMS *m*/*z* values. If several peaks were assigned the same candidate, the candidate was given the branch number.

### 2.4. Statistical Analysis

Statistical analyses were performed using gene expression profiling and metabolite analysis data obtained from three biological replicates (*n* = 3) per weaning stage. The weaning stage (before weaning, 1 week after weaning, and 2 weeks after weaning) was treated as the independent variable. For RNA-seq data, Gene Ontology and KEGG pathway enrichment analyses were conducted using the EASE score, which is used in DAVID bioinformatics. For metabolite analysis, Welch’s *t*-test was used to identify significantly altered metabolites and KEGG pathway clustering between weaning stages, with * *p*-value < 0.05, ** *p*-value < 0.01, and *** *p*-value < 0.001 to indicate statistical differences. A principal component analysis (PCA) was performed using HMT’s statistical analysis software, MasterHands (ver. 2.17.1.11, developed at Keio University, Tokyo, Japan).

## 3. Results

### 3.1. Differentially Expressed Genes in Pigs at Three Weaning Stages

**Figure 1:**
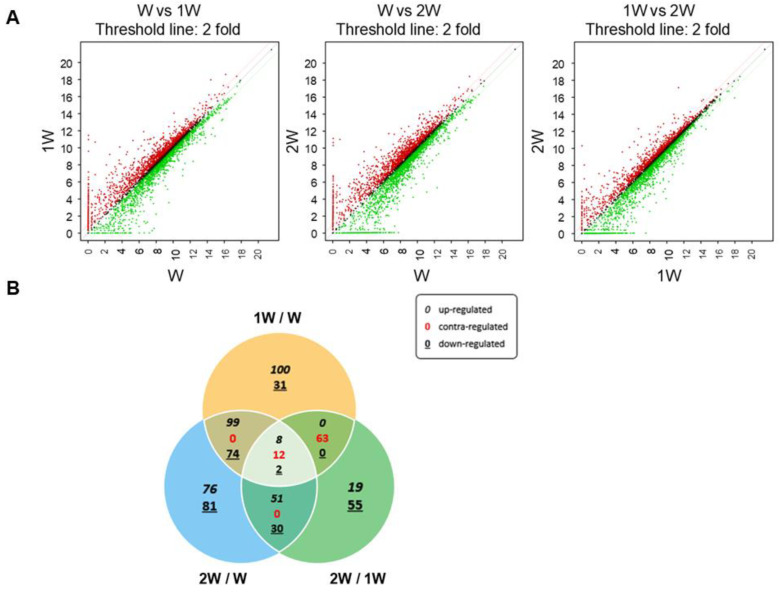
Gene expression profiling of pig intestinal tissue during weaning. (**A**) Differentially expressed genes between each weaning period, before weaning (W) vs. 1 week after weaning (1 W), W vs. 2 weeks after weaning (2 W), and 1 W vs. 2 W, are visualized using scatter plots. The red dots represent genes whose expression increased at least 2-fold, and the green dots represent genes whose expression decreased at least 2-fold. The x- and y-axes show log_2_ normalized gene expression data for each period. (**B**) Venn diagram showing the overlap of differentially expressed genes among the three comparisons: 1 W/W, 2 W/W, and 2 W/1 W.

To identify genes associated with weaning in small intestine of pigs, gene expression profiling was performed at three stages: before weaning (W), 1 week after weaning (1 W), and 2 weeks after weaning (2 W). Three comparisons were used to identify DEGs: before weaning vs. 1 week after weaning (1 W/W), 2 weeks after weaning vs. before weaning (2 W/W), and 1 week after weaning vs. 2 weeks after weaning (2 W/1 W). In gene expression profiling, genes that exhibit significant differences in expression levels between groups were defined as DEGs. Significant differences were those that satisfied the following conditions: fold changes ≥ 2, *p*-values < 0.05, and normalized data ≥ 2, and DEGs were visualized in scatter plots (Figure 1A). From a total of 3911 genes in the raw RNA-seq data, 701 DEGs related to weaning were identified (Figure 1B).

### 3.2. Functional Annotations of the Differentially Expressed Genes in the Three Weaning Stages

**Figure 2:**
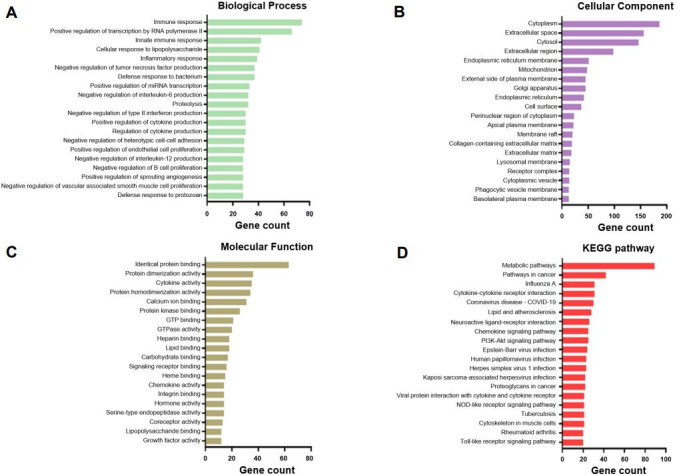
Top 20 gene Ontology (GO) and KEGG pathways of the differentially expressed genes related to weaning stress in pig intestinal tissue, including Biological Process (**A**), Cellular Component (**B**), and Molecular Function (**C**) GO category terms and KEGG pathway terms (**D**).

In the GO pathway analysis based on a total of 701 DEGs, the 20 categories with the highest gene counts were determined. Total DEGs were shown to be associated with the Biological Process terms “immune and inflammatory response,” “Proteolysis,” “regulation of cytokine production,” “negative regulation of tumor necrosis factor (TNF) production,” “negative regulation of interleukin-6 (IL-6) production,” “negative regulation of type II interferon (IFN-r) production,” “negative regulation of interleukin-12 (IL-12) production,” “negative regulation of B cell proliferation,” “negative regulation of heterotypic cell-cell adhesion,” “positive regulation of endothelial cell proliferation,” and “negative regulation of endothelial cell apoptotic process” (Figure 2A and Table S1). Cellular Component category terms included “cytoplasm,” “extracellular space,” “cytosol,” “extracellular region,” and “endoplasmic reticulum membrane” (Figure 2B and Table S2). The Molecular Function category terms showed that DEGs were involved in identical protein, lipid, calcium ion, and carbohydrate binding and cytokine, chemokine, hormone, and growth factor activity (Figure 2C and Table S3). Additionally, the KEGG pathway analysis revealed that the DEGs were related to metabolic pathways, influenza A, cytokine–cytokine receptor interactions, and proteoglycans in cancer, as well as chemokine, PI3K-Akt, NOD-like receptor (NLR), and Toll-like receptor (TLR) signaling pathways (Figure 2D and Table S4).

### 3.3. Alterations to the Porcine Intestine Metabolome During Weaning

**Figure 3:**
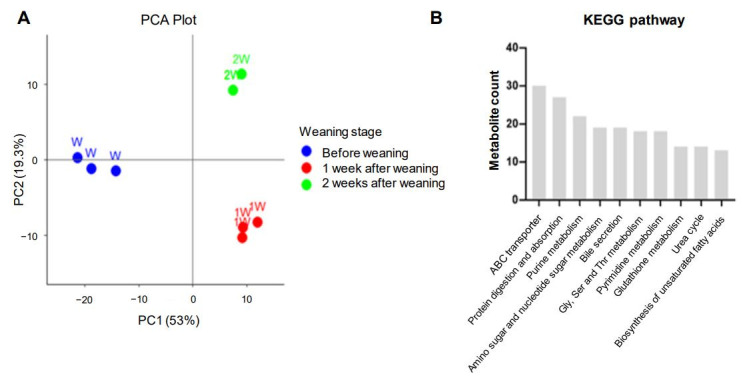
Metabolomic analysis of porcine intestinal tissue during weaning. (**A**) A PCA plot of metabolomes at different weaning stages: before weaning (W; blue dots), 1 week after weaning (1 W; red dots), and 2 weeks after weaning (2 W; green dots). (**B**) KEGG pathway analysis results for the metabolites altered by weaning stress.

To investigate metabolite changes during weaning, CE-TOFMS and LC-TOFMS analyses were performed on pig small intestine samples collected at the three weaning stages: W, 1 W, and 2 W. A total of 360 metabolites were identified based on HMT’s standard library: 170 in CE-TOFMS cation mode, 98 in CE-TOFMS anion mode, 36 in LC-TOFMS positive mode, and 56 in LC-TOFMS negative mode (Table S5). To analyze the metabolic patterns of the three stages, clustering was confirmed through PCA. The first two principal components (PC1: 52.98%, PC2: 19.28%) together explained a total of 72.26% of the variance. The plot showed dramatic separations between the three stages (Figure 3A). In addition, the top 10 KEGG pathways related to weaning were clustered to identify the associated metabolites related to weaning stress, revealing that ABC transporters, protein digestion and absorption, bile secretion, glutathione metabolism, and the biosynthesis of unsaturated fatty acids were associated with weaning stress (Figure 3B and Table S6).

## 4. Discussion

In the life of mammals, weaning is a stressful alteration requiring challenging nutritional and immune system adjustments [16,27]. If these challenges become overwhelming, disease, anorexia, malabsorption, and growth retardation can result [13,28]. These weaning-mediated alterations are especially important issues in livestock industries, which aim for maximum productivity [8,29]. The small intestine plays the major role in maintaining homeostasis in response to these challenges [21,30,31]. It is vital for nutrient transfer and is a major barrier regulating movement in and out of the body, functions that cooperatively ensure proper nutrient utilization and defense against noxious factors, making the small intestine particularly important during the weaning transition [22,25,32]. Previous studies have shown that weaning hinders intestinal morphological, structural, and functional integrity and diverse barrier development [33,34]. Studies investigating changes to biomolecule markers during weaning have identified numerous affected regulatory genes and metabolites, including growth factors, hormones, digestive enzymes, immunocytes, cytokines, and immunoglobulins [35,36,37]. Despite these findings, the biomolecule-mediated mechanisms acting during the transition period remain complex and unknown.

Thus, this study analyzed the transcriptomes of porcine intestinal tissues before weaning, 1 week after weaning, and 2 weeks after weaning. We identified 701 DEGs related to weaning. Based on these DEGs, various GO and KEGG pathway terms associated with overall intestinal health were identified. As an immune organ, the small intestine is the first barrier that pathogens encounter, putting it at the center of self-immune regulation and making it the first responder to antigens [31,38]. Our data revealed that a number of immune system-related terms were enriched during weaning. Previous studies have shown that endogenous and exogenous antigens encountered during weaning can induce various immunological activities and simultaneously increase susceptibility to disease [26,39,40]. That is, piglets, having immature immune systems, are susceptible to diseases due to excessive immune responses or failures in immune defense.

Additionally, GO and KEGG pathways associated with cell proliferation, cell adhesion, and apoptosis, as well as various metabolic binding and activity pathways, were affected during weaning. Under the stresses connected to weaning, the small intestine is structurally damaged by unavoidable novel foreign substances, resulting in the loss, atrophy, shedding, and hyperplasia of villi and crypts, which makes functional intestinal cells, such as epithelial, endocrine, Paneth, mucosal, and goblet cells, vulnerable [25,41,42]. Research is gradually showing that gene expression and molecular mechanisms in the intestine cause dysfunctional proliferation, apoptosis, and permeability during weaning stress [10,43,44]. Moreover, these negative effects are directly related to and interact with a diverse array of metabolites, creating a complex biological network of effects. It is becoming clear that integrated analyses and approaches will be needed to enhance our understanding of weaning-mediated intestinal alterations.

We also performed a metabolome analysis using CE-TOFMS and LC-TOFMS and identified 360 metabolites that may be associated with weaning stress in the porcine intestine. In a PCA plot, metabolomes from before weaning, 1 week after weaning, and 2 weeks after weaning formed distinct groups, suggesting that each stage exhibits a different metabolic response or that unique metabolites are regulated during each stage. Furthermore, based on a KEGG pathway analysis of metabolites, we showed that weaning affects ABC transporters, protein digestion and absorption, various biological compounds’ metabolisms, and the urea cycle. When cells are damaged, the body’s metabolic system enhances energy metabolism to maintain homeostasis [45,46]. Purine and pyrimidine play important roles in DNA and RNA synthesis, and amino and nucleotide sugars are incorporated into many cellular structures [47,48]. Then, amino acids supplied through protein digestion and absorption are involved in protein synthesis for cell maintenance and survival [49]. However, excessive energy production causes reactive oxygen species accumulation, and therefore, ABC transporters, Gly-Ser-Thr metabolism, and glutathione metabolism are activated to maintain redox balance, and the urea cycle is required to maintain balance in the body by excreting waste products [50,51,52].

To maximize pig production, the porcine industry must strive to minimize the suffering experienced by piglets during the weaning transition. A comprehensive understanding, and thus the meticulous investigation, of weaning stress is required to overcome digestive, immune, structural, and molecular disorders in the small intestine that occur during weaning. The current study speculates that the intestinal response to weaning in piglets is related to the activation of energy metabolism to increase cell survival and maintain normal homeostasis in the face of weaning-induced alterations, which may be closely related to the adverse effects of weight loss and increased disease susceptibility in newly weaned piglets.

The purpose of this study is to identify changes in gene and metabolite expression related to weaning stress in the small intestine of piglets. Therefore, it is expected that there will be limitations in identifying and analyzing specific genes and metabolites at each time period. In future studies, it is expected that it will be necessary to identify significant genes and metabolites at each weaning time period, discover biomolecules that enhance the biological health of weaned piglets, and develop feed additives that mitigate weaning stress.

## 5. Conclusions

We identified 701 DEGs associated with weaning stress in the porcine intestine that may influence the molecular mechanisms underlying the deleterious effects of weaning. Furthermore, we showed that metabolites related to damage and damage recovery were also conspicuous in the metabolomes of the post-weaning stages. Our results provide insights that better our understanding of weaning stress and evidence that can be used for the development of metabolite-based biomarkers distinguishing weaning periods, both of which will benefit the porcine industry.

## Data Availability

The original data of this study is included in this paper. Further inquiries can be directed to the corresponding author.

## Supplementary Materials

The following supporting information can be downloaded at https://www.mdpi.com/article/10.3390/genes16070727/s1: Table S1: Biological process of total differentially expressed genes, Table S2: Cellular component of total differentially expressed genes, Table S3: Molecular function of total differentially expressed genes, Table S4: KEGG pathway of total differentially expressed genes, Table S5: Metabolites identified by capillary electrophoresis time-of-flight mass spectrometry and liquid chromatography time-of-flight mass spectrometry, and Table S6: KEGG pathway clustering of identified metabolites.
